# Mesenchymal marker expression is elevated in Müller cells exposed to high glucose and in animal models of diabetic retinopathy

**DOI:** 10.18632/oncotarget.13945

**Published:** 2016-12-15

**Authors:** Ti Zhou, Di Che, Yuqing Lan, Zhenzhen Fang, Jinye Xie, HaiJun Gong, ChaoYang Li, Juan Feng, Honghai Hong, Weiwei Qi, Caiqi Ma, Zhonghan Yang, WeiBin Cai, Jun Zhong, Jianxing Ma, Xia Yang, Guoquan Gao

**Affiliations:** ^1^ Program of Molecular Medicine, Affiliated Guangzhou Women and Children's Hospital, Zhongshan School of Medicine, Sun Yat-Sen University, Guangzhou, China; ^2^ Department of Biochemistry, Zhongshan School of Medicine, Sun Yat-sen University, Guangzhou, China; ^3^ Department of Ophthalmology, Second Affiliated Hospital, Sun Yat-Sen University, Guangzhou, China; ^4^ State Key Laboratory of Ophthalmology, Zhongshan Ophthalmic Center, Sun Yat-sen University, Guangzhou, China; ^5^ Guangdong Engineering and Technology Research Center for Disease-Model Animals, Sun Yat-Sen University, Guangzhou, China; ^6^ Department of Physiology, University of Oklahoma, Health Sciences Center, Oklahoma City, Oklahoma, USA; ^7^ Key Laboratory of Functional Molecules from Marine Microorganisms (Sun Yat-sen University), Department of Education of Guangdong Province, Guangzhou, China; ^8^ China Key Laboratory of Tropical Disease Control (Sun Yat-sen University), Ministry of Education, Guangzhou, China

**Keywords:** diabetic retinopathy, mesenchymal markers, hyperglycemia, müller cells

## Abstract

Müller cells are retinal glial cells and exhibit a fibroblast-like phenotype and ability to migrate in diabetic retinopathy (DR). However, expression of mesenchymal markers, which promote fibrosis in various organs, has not been characterized in the diabetic retina. We examined changes in the expression of these markers in Müller cells exposed to high glucose and in animal models of diabetic retinopathy. High glucose conditions increased mesenchymal maker expression and migration in Müller cells. Snail, N-cadherin, Vimentin, β-catenin, and α-smooth muscle actin (α-SMA) levels were all dramatically increased in retinas from humans with diabetic retinopathy (DR) and from DR mouse models. In addition, Snail overexpression increased the expression of connective tissue growth factor (CTGF) and fibronectin, while Snail knockdown attenuated high glucose-induced increases in fibronectin and CTGF expression. These results demonstrate for the first time that mesenchymal markers are upregulated in retinas from a diabetic mouse model, and that Snail and N-cadherin levels are also increased in Müller cells exposed to high glucose. This suggests mesenchymal proteins may play a crucial role in the development of DR.

## INTRODUCTION

Diabetic retinopathy (DR) is a severe complication of diabetes and the leading cause of blindness among working adults worldwide [1]. DR is classified as either non-proliferative (non-PDR) or proliferative (PDR) [2]. The main pathogenic features of PDR are preretinal neovascularization and the formation of fibrovascular membranes at the vitreoretinal interface. The presence of fibrovascular tissue often results in severe visual impairment due to vitreous hemorrhages and/or tractional retinal detachment [3]. Although retinal neovascularization has been considered the main characteristic of PDR, the fibrogenic process that occurs after new vessels are formed results in a traction force and, eventually, retinal detachment, suggesting that PDR is a fibrotic disease as well [4].

The pathogenesis of DR is extremely complex and involves many different cells, molecules, and factors. Endothelial, Müller, ganglion, and pigment epithelial cells are implicated in DR pathogenesis [1]. Müller cells express and secrete growth factors and cytokines that lead to retinal neuron and capillary cell dysfunction [5]. Immunohistochemical studies revealed that Müller cells are present in the diabetic epiretinal membrane [6–8]. In addition, Müller cells can exhibit characteristics of fibroblast cells and can generate tractional force in response to cytokines [6, 9].

Specific mesenchymal markers are involved in kidney, lung, and liver fibrosis [10–13]. For example, increased vimentin levels in tubular epithelial cells correlated with declining renal function in diabetic patients [14], and snail activation is sufficient to induce kidney fibrosis in adult transgenic mice [15]. In addition, a-SMA is considered the most reliable marker of myofibroblastic differentiation [16]. N-cadherin and Snail promote migratory and invasive cellular phenotypes [17, 18]. This evidence indicates that mesenchymal proteins play a crucial role in cellular dysfunction and in the development of tissue fibrosis. Because Müller cells are crucial for traction-induced retinal detachment [9], in this study we examined changes in mesenchymal marker expression in Müller cells exposed to high glucose conditions and in the diabetic retina and the role of these markers in DR pathogenesis.

## RESULTS

### Expression of mesenchymal markers in PDR epiretinal membranes

**Figure 1:**
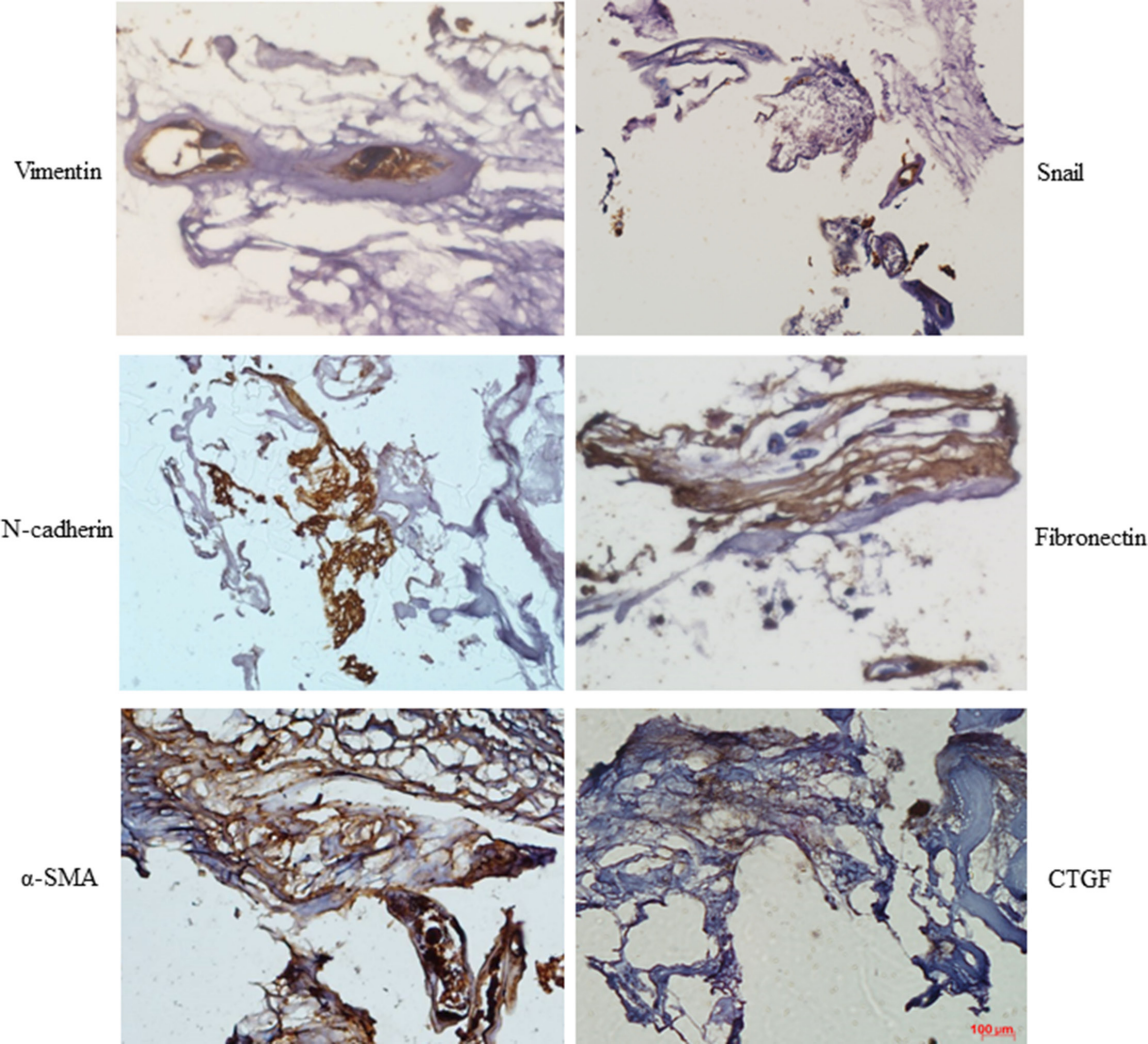
Mesenchymal marker expression was increased in PDR membranes Sections of epiretinal membrane from PDR patients were immunostained with mesenchymal marker antibodies. Diaminobenzidine (brown color) was used to visualize staining. Representative images of retina showing Vimentin, N-cadherin, α-SMA, Snail, fibronectin, and connective tissue growth factor (CTGF) staining in epiretinal membranes from PDR patients (*n* = 3); scale bar: 100 μm.

Immunostaining revealed that vimentin, N-cadherin, α-SMA, and Snail were expressed in epiretinal membranes from PDR patients. Fibronectin and connective tissue growth factor (CTGF), which are important profibrotic growth factors in DR, were also expressed in epiretinal membranes from PDR patients (Figure 1).

### Mesenchymal markers were up-regulated in the retinas of STZ-diabetic mice

**Figure 2:**
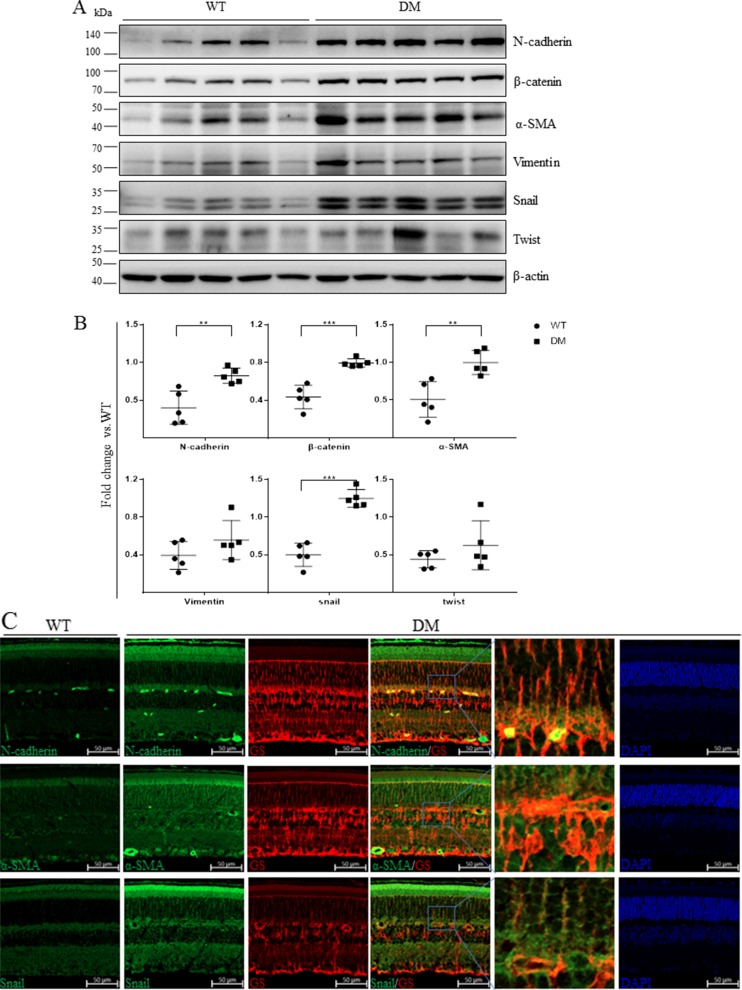
Mesenchymal marker expression was increased in the retinas of STZ-diabetic mice (**A**) The expression of mesenchymal markers in non-diabetic mice and mice with STZ-induced diabetes was measured using Western blots (*n* = 5). Each lane represents an individual animal. (**B**) Quantification of the bands from WT and DM mice in Figure 2A (**p < 0.01, *p < 0.05, *n* = 5). β-actin served as a loading control. (**C**) Representative retinal images showing increases in the colocalization of N-cadherin, α-SMA, and Snail (green) with the Müller cell marker GS (red) in STZ-diabetic mice compared to control mice; scale bar: 50 μm.

To confirm these results in DR animal models, we examined mesenchymal marker levels in the retinas of mice with STZ-induced diabetes (STZ-diabetic) mice 16 weeks after the onset of diabetes. As shown in Figure 2A and 2B, N-cadherin, β-catenin, α-SMA, and Snail levels increased substantially in diabetic retinas compared to those from age-matched wild-type controls. Ocular sections from the eyes of STZ-diabetic and control mice were then stained with specific antibodies for mesenchymal markers and visualized using immunofluorescence. N-cadherin, α-SMA, and Snail levels were higher in retinas from diabetic mice, and these mesenchymal markers partially colocalized with the Müller cell marker GS (Figure 2C).

### Mesenchymal markers were up-regulated in the retinas of db/db mice

**Figure 3:**
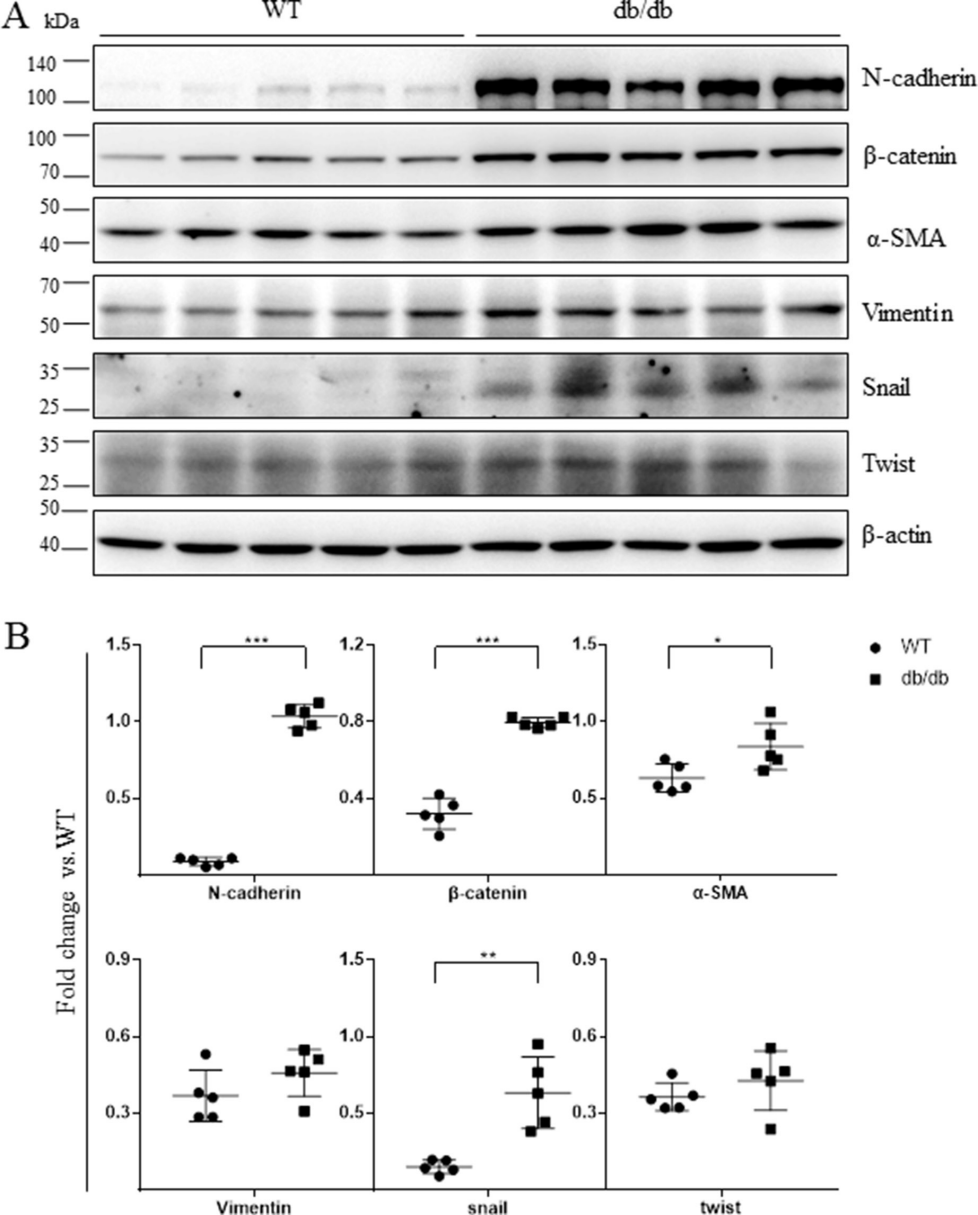
Mesenchymal marker expression was increased in the retinas of db/db mice (**A**) The expression of mesenchymal markers in 16-week-old wild-type mice and db/db mice was examined using Western blots (*n* = 5). Each lane represents an individual animal. (**B**) Quantification of the bands from WT and db/db mice in Figure 3A (**p < 0.01, *p < 0.05, *n* = 5). β-actin served as a loading control.

Next, we evaluated mesenchymal marker levels in the retinas of 16 week-old db/db mice, a genetic model of type 2 diabetes. As in the STZ-diabetic mice, N-cadherin, β-catenin, α-SMA, and Snail levels were increased in the retinas of db/db mice (Figure 3A, 3B).

### High glucose conditions promote mesenchymal activation in Müller cells

**Figure 4:**
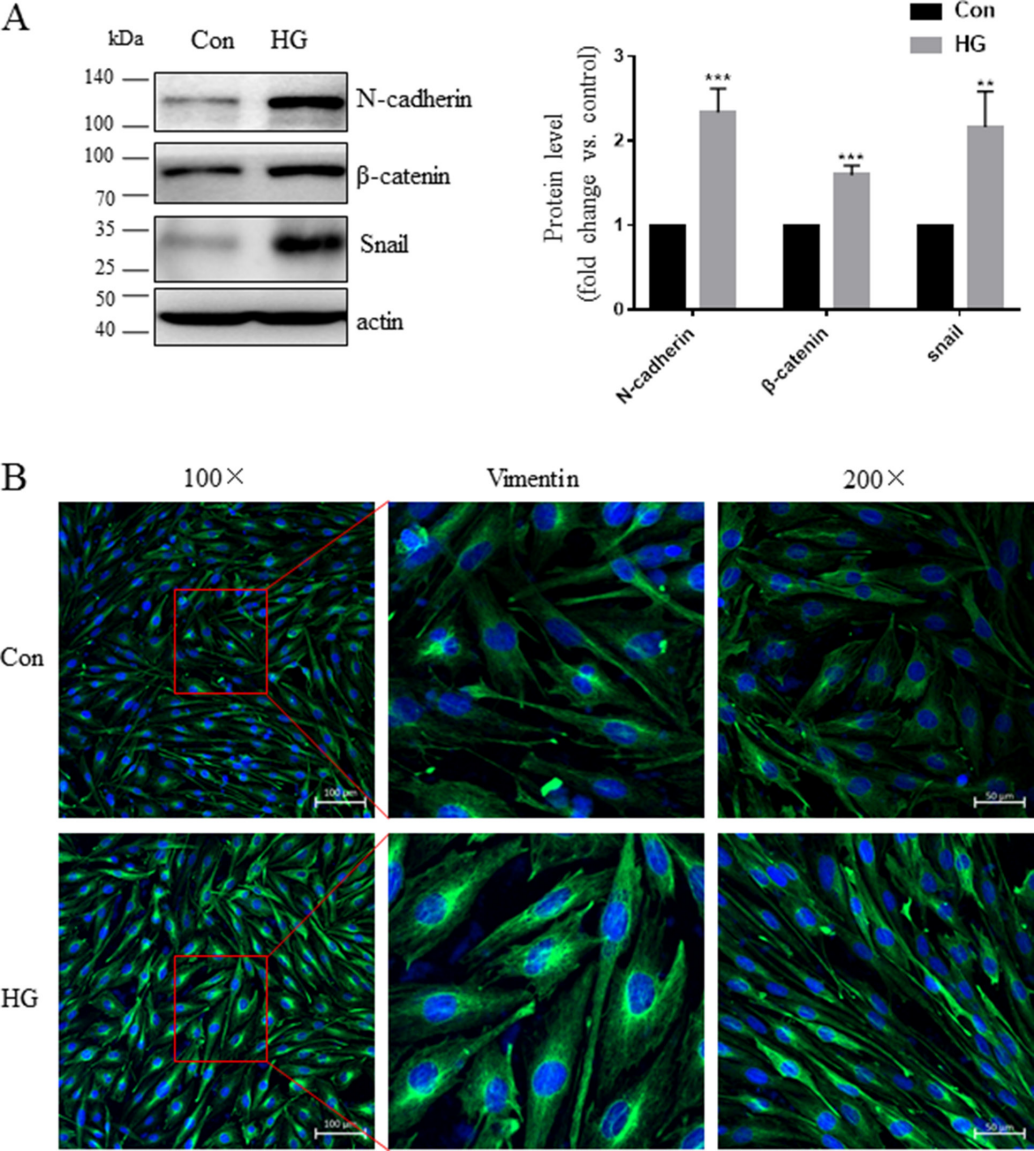

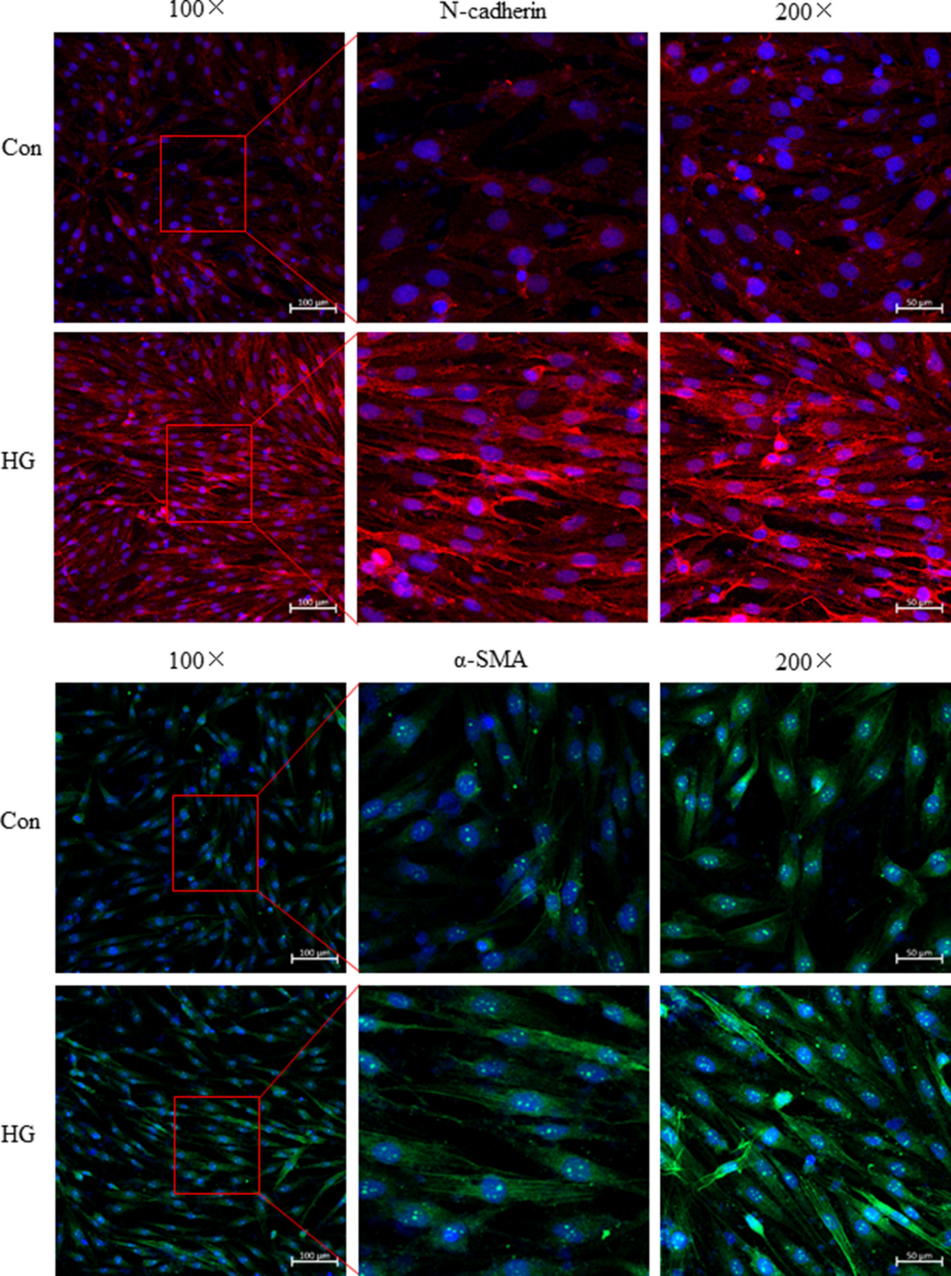

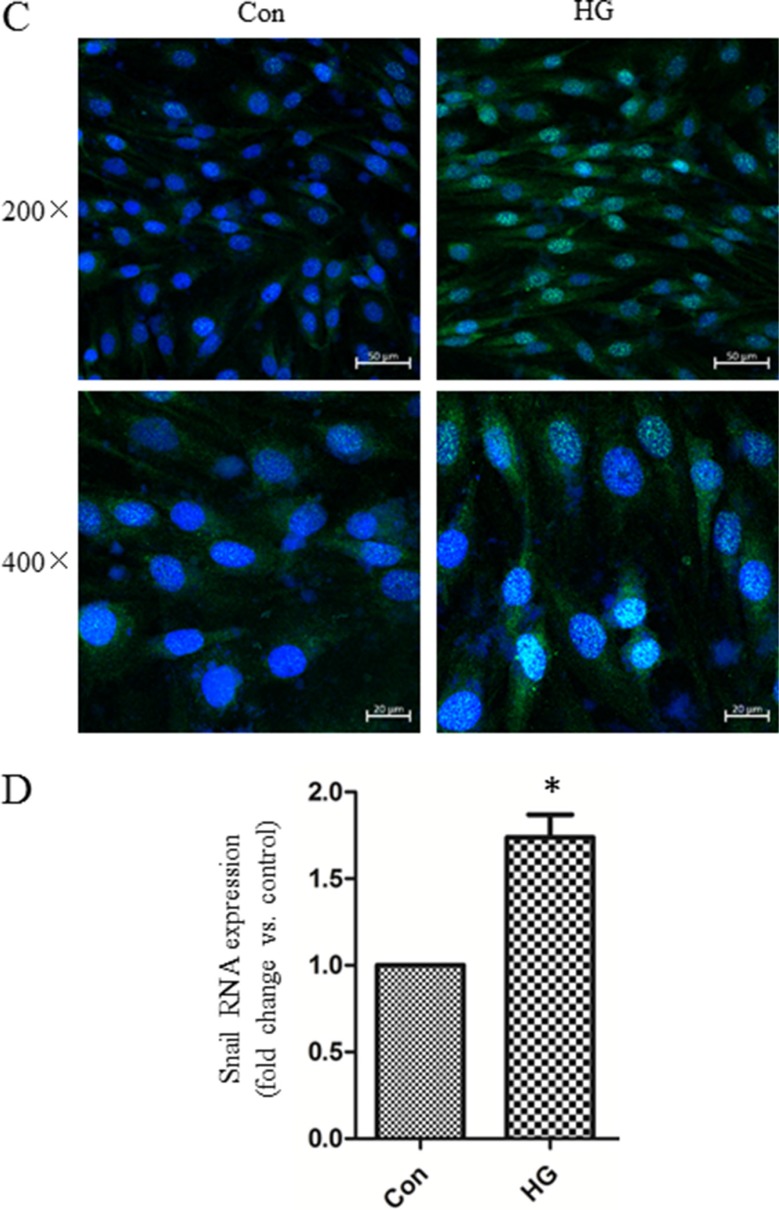
High glucose conditions increased mesenchymal marker expression in Müller cells (**A**) Representative Western blots showing N-cadherin, β-catenin, and Snail expression in cells treated with 30 mM D-glucose (HG) or L-glucose as a control for 48 hours. The bands were quantified relative to β-actin (mean ± s.d., **p < 0.01, *p < 0.05, *n* = 3). (**B**–**C**) Cells were grown on glass cover slips for 24 hours, starved for 24 hours, and then incubated with HG or L-glucose as a control for 48 hours. Vimentin, N-cadherin, α-SMA, and Snail expression as indicated by confocal microscopy were increased in HG cells compared to control cells; scale bar: 50 μm. (**D**) Real-time PCR analysis of SNAIL gene expression in cells after 48 hours of incubation with HG or L-glucose as a control. Means ± s.d. are shown, *p < 0.05.

**Figure 5:**
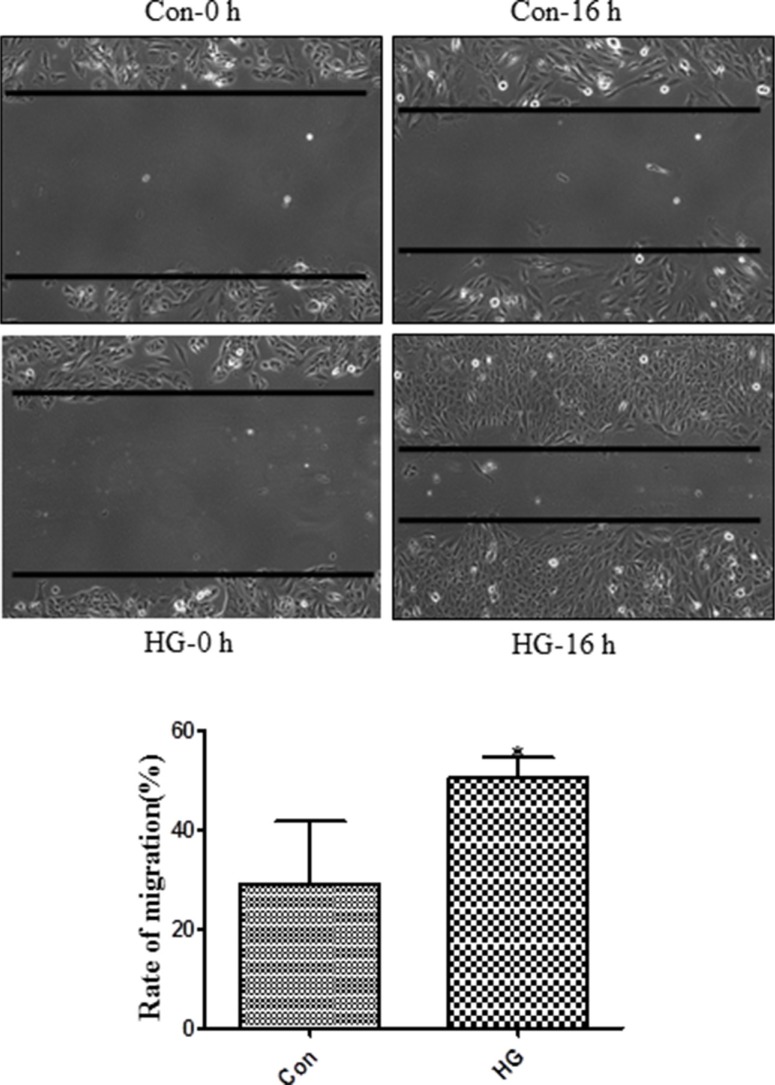
High glucose treatment promoted migration in Müller cells Müller cell monolayers were grown in six-well plates to approximately 80% confluence. Cells were starved in DMEM with 2% FBS for 24 hours and then treated with L-glucose or D-glucose (30 mM) for 48 hours. Migration distances were measured after a scratch was applied to the monolayer. These experiments were conducted independently in triplicate. Means ± s.d. are shown; *p < 0.05 compared to L-glucose control.

First, we examined mesenchymal marker levels in Müller cells exposed to high glucose conditions. As shown in Figure 4A, compared to the L-glucose osmotic control treatment, 48 h of exposure to 30 mM glucose increased N-cadherin and β-catenin levels. Vimentin, N-cadherin, and α-SMA immunostaining were stronger in high glucose-treated cells than in control cells (Figure 4B). In addition, high glucose conditions induced the translocation of Snail to the nucleus compared to control cells (Figure 4C) and increased mesenchymal marker mRNA and protein levels in Müller cells (Figure 4A, 4D). Exposure to high glucose also increased Müller cell migration compared to the control group (Figure 5).

### Snail increased cytokine expression in Müller cells

**Figure 6:**
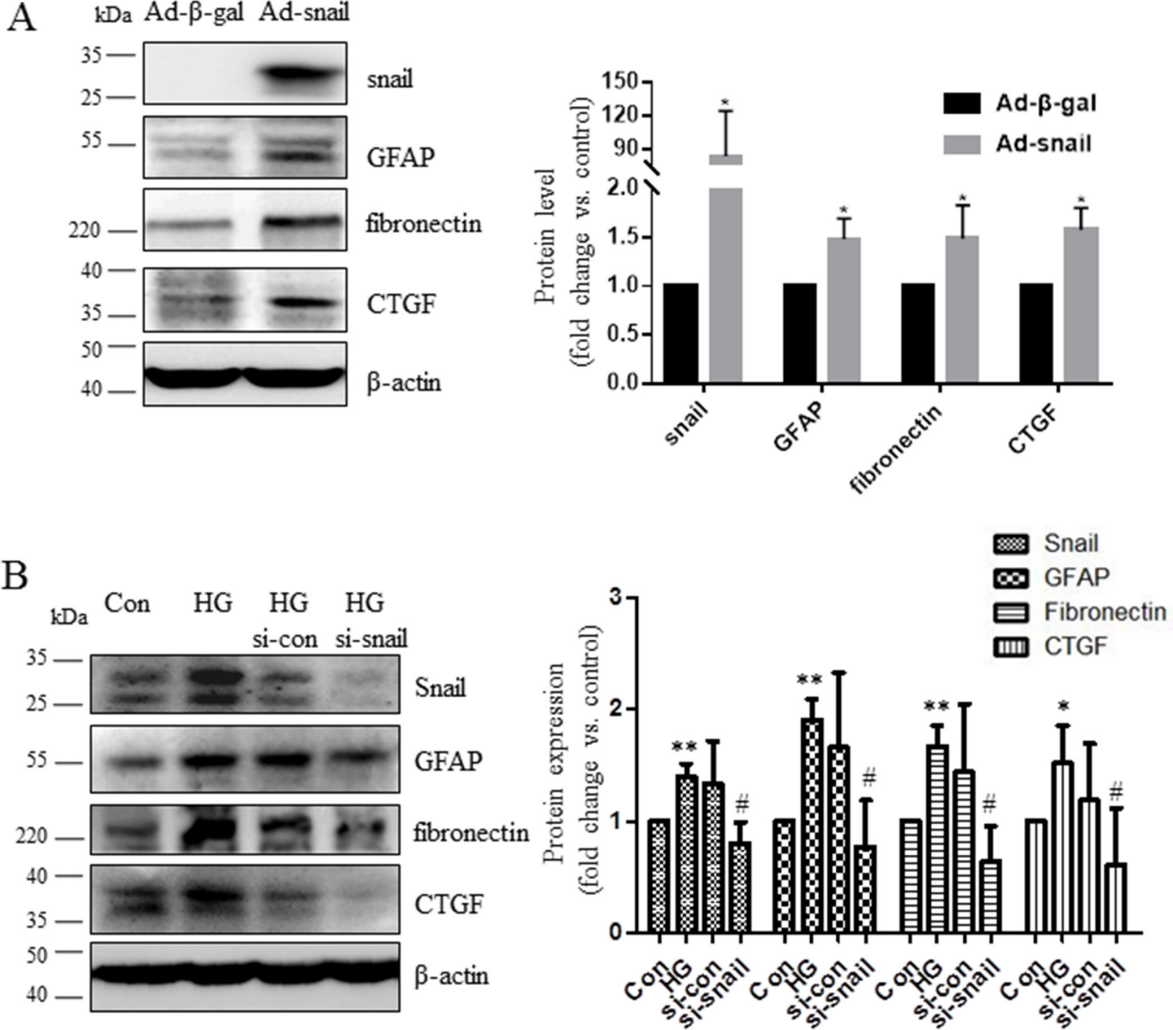
Snail increased CTGF and fibronectin expression in Müller cells (**A**) Müller cells were infected with adenovirus expressing Snail (Ad-Snail) or control adenovirus expressing β-galactosidase (Ad-β-gal) with an MOI of 20 for 48 hours. (**B**) Müller cells were transfected with control siRNA or Snail-siRNA for 24 hours then incubated in high glucose conditions for additional 48 hours. Total cellular proteins were immunoblotted with antibodies for GFAP, fibronectin, and CTGF. Blots are representative of three independent experiments. The bands were quantified relative to β-actin (mean ± s.d., **p < 0.01, *p < 0.05, *n* = 3).

Connective tissue growth factor (CTGF) is an important profibrotic growth factor that induces extracellular matrix (ECM) production and angiogenesis [19]. CTGF is upregulated in humans with DR and in rodent models of DR [20, 21], and its expression is induced by exposure to high glucose conditions [20, 22]. Fibronectin expression is also increased in the retinal tissues of diabetic rats [23] and in vitreous specimens from patients with PDR [24–26]. In order to investigate the role of mesenchymal markers in DR pathogenesis, Müller cells were infected with adenovirus expressing Snail (Ad-Snail) or with adenovirus expressing β-galactosidase (Ad-β-gal) at the same multiplicity of infection (MOI) as a control. Snail expression increased dramatically, while fibronectin and CTGF expression increased to a lesser degree, in Ad-Snail cells compared to those infected with Ad-β-gal (Figure 6A). Meanwhile, compared to control cells, silencing Snail expression in Müller cells attenuated glucose-induced increases in CTGF and fibronectin expression (Figure 6B). In addition, Snail induced glial fibrillary acidic protein (GFAP) expression in Müller cells (Figure 6). These data suggest that Snail might contribute to fibrosis/gliosis and PDR membrane formation by upregulating the expression of key cytokines.

## DISCUSSION

In this study, we systematically evaluated mesenchymal marker expression in the retinas of humans with DR and in animal models of both type 1 and types 2 diabetes. We found that mesenchymal marker levels were increased in the retinas of humans with DR and of STZ-induced and db/db diabetic mice. In addition, hyperglycemia induced the expression of mesenchymal markers and fibrogenic factors in Müller cells. Moreover, Snail further increased the high glucose-induced expression of fibrogenic factors in Müller cells.

We found that N-cadherin, α-SMA, Snail, fibronectin, and CTGF were expressed in the epiretinal membranes (ERM) of patients with DR (Figure 1), suggesting that these mesenchymal markers are involved in the development of ERM and DR. We then examined the expression of these markers in animal models of DR and found that N-cadherin, β-catenin, α-SMA, and Snail were all up-regulated in the retinas of both STZ-induced and db/db diabetic mice (Figures 2, 3). Similar changes in mesenchymal marker expression were also observed in the retinas of STZ-induced diabetic rats (Supplementary Figure S1). These data indicate that the development of DR associated with either type 1 or type 2 diabetes is characterized by up-regulation of mesenchymal markers.

Retinal Müller cells are particularly susceptible to diabetes-induced damage and play a crucial role in the initiation and progression of DR [27]. Epiretinal membrane formation is associated with gliosis, fibrosis, and migration of Müller cells [28]. Here, we found that diabetes upregulated N-cadherin, α-SMA, and Snail levels and that these proteins co-localized with the Müller cell marker GS (Figure 2C). We therefore examined whether hyperglycemia was sufficient for increasing mesenchymal marker levels in retinal Müller cells. As was the case in a previous study [29], we found that high glucose conditions increased α-SMA, fibronectin, and connective tissue growth factor (CTGF) levels in Müller cells. Furthermore, we found that high glucose conditions also increased N-cadherin, β-catenin, Vimentin, and Snail levels (Figure 4). High glucose conditions also promoted Müller cell migration in a wound healing assay (Figure 5). Elevated Snail levels are associated with increased motility as well [30–32], and we observed here that Snail stimulated migration in Müller cells (data not shown). These observations indicate that mesenchymal markers might be responsible for cellular dysfunction during hyperglycemia and for the development of DR.

CTGF, which is secreted primarily by activated Müller cells, promotes fibrosis in diabetic retinopathy [33] and eventually activates downstream fibronectin production [34] and fibroblast proliferation [35]. However, previous studies have not examined the link between Snail expression and fibrogenic factors in Müller cells. Here, we demonstrated for the first time that Snail increased the expression of CTGF and fibronectin, which were the main fibrogenic factors produced by Müller cells (Figure 6). Notably, Snail also increased levels of GFAP, which is the most widely-used indicator of changes in Müller cells triggered by environmental stress [9]. It is possible that Snail causes abnormal secretion of cytokines from Müller cells that directly act on the retina. Müller cells have many functions in both normal and disease states [9], and Snail may participate in other retinal processes besides fibrosis. Additional studies are needed to further characterize the effects of Snail expression in the retina. Increased expression of mesenchymal markers has also been associated with phenotypic changes in endothelial cells and with dysfunctions related to diabetic retinopathy [36]. Our data expand upon previous studies to characterize the roles of specific mesenchymal makers in the diabetic retina.

In summary, we demonstrated for the first time that specific mesenchymal markers are upregulated in the diabetic retina and that Snail and N-cadherin levels were increased in Müller cells exposed to high glucose conditions. These findings shed new light on the pathogenic role of mesenchymal proteins in DR.

## MATERIALS AND METHODS

### Materials and antibodies

L-glucose, D-glucose, and streptozocin were purchased from Sigma (St. Louis, MO, USA). α-SMA (A2547), β-actin (A5411), and Twist (T6451) antibodies were purchased from Sigma-Aldrich (St Louis, MO, USA). Antibodies against Vimentin (550513), N-cadherin (610920), and fibronectin (610077) were obtained from BD (Franklin Lakes, NJ, USA). Antibodies for Snail (3879S), β-catenin (9582S), and Glial Fibrillary Acidic Protein (GFAP, 3670S) were from Cell Signaling Technology (Danvers, MA, USA). CTGF (ab6922) and Glutamine synthase (GS, ab176562) antibodies were purchased from Abcam (Cambridge, MA, USA). Glutamine synthase (GS, MAB302) antibody was from Millipore (Billerica, MA, USA). Goat Anti-mouse(PI-2000) and anti-rabbit (PI-1000) horseradish peroxidase (HRP)-conjugated secondary antibodies were from Vector Laboratories (Burlingame, CA, USA). Alexa Flour 488 goat anti-rabbit/anti-mouse (A21206/A21202) and Alexa Fluor 594 goat anti-rabbit/anti-mouse (A21207/A21203) antibodies and 4’,6-Diamidino-2-phenylindole (DAPI, D1306) were from Life Technology (St. Louis, MO, USA).

### Epiretinal membrane specimens

Human ocular samples were collected in strict agreement with guidelines approved by the Second Affiliated Hospital of Sun Yat-Sen University, and each participant gave written informed consent. Epiretinal membranes were obtained during vitreoretinal surgery for the treatment of PDR. All experiments were approved by the Medical Ethics Committee of Sun Yat-sen University (Guangzhou, Guangdong, China).

### Animal studies

The care, use, and treatment of all animals were conducted in strict agreement with the guidelines of the Association for Research in Vision and Ophthalmology Statement for the Use of Animals in Ophthalmic and Visual Research and were approved by the institutional animal care and use committees at Sun Yat-sen University. C57BL/6 J mice were purchased from the Laboratory Animal Center of Guangdong, China under the animal license number *SCXK (YUE)2008–0020*. Sixteen week-old male db/db mice (BKS.Cg-D ock7m +/+Leprdb/J) were purchased from the Laboratory Animal Center of Nanjing, China. Mice were housed in groups of five and allowed to acclimate to the housing facility (specific pathogen-free) for five days. The housing facility was maintained at a temperature of 21°C ± 2°C, humidity of 55% ± 10%, lighting of 350 lux (at bench level), and a 12:12 light:dark cycle with lights on at 0700 and off at 1900. Animals were housed in 595 × 380 × 200 mm cages and given access to mouse maintenance food and water. Environmental enrichment included bedding (LBS, Litaspen Premium B6 grade), one mouse hut (Bio-Serv, cat# K3272), one 10 × 10 × 50 mm aspen chew block (LBS, cat# 011590), and one handful of paper wool nesting material (LBS, cat# 033801). Animals were monitored twice daily for health status; no adverse events were observed. To induce diabetes, 8-week-old C57BL/6 J male mice were given five consecutive intraperitoneal injections of streptozotocin (STZ; 50 mg/kg body wt/day) (Sigma-Aldrich) or vehicle as a control. Blood glucose levels were measured 48 hours after the STZ injection; only animals with glucose levels > 350 mg/dL were considered diabetic. Sixteen weeks after STZ injection, mice were euthanized with 10% chloral hydrate, and eyes were harvested for analyses. All experimental protocols were approved by the Animal Care and Use Committee of Sun Yat-sen University (Guangzhou, Guangdong, China).

### Cell culture

Müller cells (MIO-M1) were cultured in DMEM containing 10% heat inactivated fetal bovine serum (FBS) and 1% antibiotics. Cells were maintained at 37°C in a humidified atmosphere with 5% CO_*2*_.

### Immunohistochemistry

Immunohistochemistry was performed as described previously [37]. For paraffin embedded human epiretinal membrane sections (5 μm), fibronectin, vimentin, and α-SMA antibodies were used at dilutions of 1:400, and the Snail and N-cadherin antibodies were used at dilutions of 1:50 and 1:100, respectively; all antibodies were incubated overnight. After thorough washes with phosphate-buffered saline (PBS), immunostaining was visualized using a kit (Vectastain ABC; Vector Laboratories, Burlingame, CA, USA) according to the manufacturer's protocol.

### Immunofluorescent staining

Immunofluorescent staining was performed as described previously [38]. For frozen embedded mice retina sections (5 μm), Snail and GS (MAB302) primary antibodies were used at dilutions of 1:50 and 1:400, respectively, and N-cadherin, α-SMA, and GS (ab176562) antibodies were used at dilutions of 1:200. For the immunocytochemistry assay, cells were grown to 60% confluence in four-well glass slide chambers and treated with 30 mM (high) glucose for 48 hours. The cells were then incubated with Vimentin, N-cadherin, and α-SMA primary antibodies at dilutions of 1:200 overnight at 4°C, followed by incubtion with Alexa Flour 488/Alexa Flour 594 goat anti-rabbit/anti-mouse secondary antibodies at dilutions of 1:200 for 1 hour. Slides were prepared with a mounting medium containing DAPI to counterstain the nucleus.

### Western blot analysis

Retinal tissue and cells were lysed for total protein extraction using RIPA buffer. Protein concentrations were determined using a Bio-Rad DC protein assay kit (Bio-Rad Laboratories) according to the manufacturer's protocol. Equal amounts of protein were resolved by SDS-PAGE and transferred to a PVDF membrane (Bio-Rad, Hercules, CA). After blocking with 5% nonfat dry milk in Tris-buffered saline Tween-20 (TBST) for 1 hour, the membrane was incubated overnight at 4°C with various primary antibodies. After washing with TBST, the membrane was incubated with the appropriate secondary antibody for 2 hours. The membrane was washed again with TBST, and immunoblots were then developed with enhanced chemiluminescent reagents from Pierce according to the manufacturer's instructions. Images were taken using ImageQuant Las 4000mini (GE) and densitometry was performed using ImageJ software and normalized to β-actin levels.

### Real-time PCR

Total RNA was extracted from cultured cells using Trizol reagent (Invitrogen, CA, USA) according to the manufacturer's instructions. Total RNA (500 ng) was used for reverse transcription using PrimeScript^®^ RT reagent Kit (Perfect Real Time) (Takara Bio Inc., Japan). The cDNA was used for quantitative real-time PCR analysis using SYBR^®^ Premix Ex Taq^™^ (Perfect Real Time) (Takara Bio Inc., Japan) and the capillary-based Light Cycler^®^ 2.0 System (Roche Diagnostics Corporation, Indianapolis, IN, USA). The specificity of the amplification reactions was confirmed by melting curve analysis. All expression data were normalized to β-actin. The data were quantified using the comparative threshold cycle (Ct) method for relative gene expression. The human Snail primer sequences are as follows: forward, TGCGCTACTGCTGCGCGAAT; reverse, GGGCTGCTGGAAGGTAAACTCTGGA.

### Wound healing assay

Cells were seeded in each well of a six-well culture plate and cultured for 24 hours until they reached approximately 80% confluence. Cells were starved in DMEM for 24 hours and then treated with L-glucose as a control or with D-glucose (30 mM) for 48 hours. The cells were then wounded by scratching; images of the wells were taken at the indicated times after wounding. Cell migration rates were calculated as the distance that cells traveled into the cell-free space from the wound edge.

### RNA interference

Oligonucleotides matching selected regions of the human Snail sequence and negative control scrambled siRNAs were purchased from Ribobio (Guangzhou, China). Cells were transfected with siRNA oligonucleotides at a final concentration of 100 nM using HiPerFect (QIAGEN, Carson City, CA) according to the manufacturer's instructions. Cells were transfected with siRNA oligonucleotides for 24 hours and then incubated in high glucose conditions for additional 48 hours.

### Construction of the Snail-expressing adenovirus

Purified and titered adenoviruses expressing Snail (Ad-Snail) or β-galactosidase (Ad-β-gal) were generated by Cyagen (Guangzhou, China) using an adenoviral vector system (AdenoVator; Qbiogene, Irvine, CA). Cells were cultured to 50% to 80% confluence and infected with virus at an MOI of 20. After 48 hours, cells were harvested and protein expression was examined using Western blots.

### Statistical analysis

Data are presented as means ± sd. Two-tailed paired Student's *t*-tests were used for comparisons; *p* < 0.05 was considered statistically significant.

## SUPPLEMENTARY MATERIALS FIGURES AND TABLES


