# Supplementary material for: Mesenchymal marker expression is elevated in Müller cells exposed to high glucose and in animal models of diabetic retinopathy
Source: Oncotarget. 2016 Dec 15;8(3):4582–94. doi: 10.18632/oncotarget.13945 (PMC5354856; doi:10.18632/oncotarget.13945)
Supplement: Supplementary file 1 [file oncotarget-08-4582-s001.pdf]

Mesenchymal marker expression is elevated in Müller cells exposed to high glucose and in animal models of diabetic retinopathy

Supplementary Materials

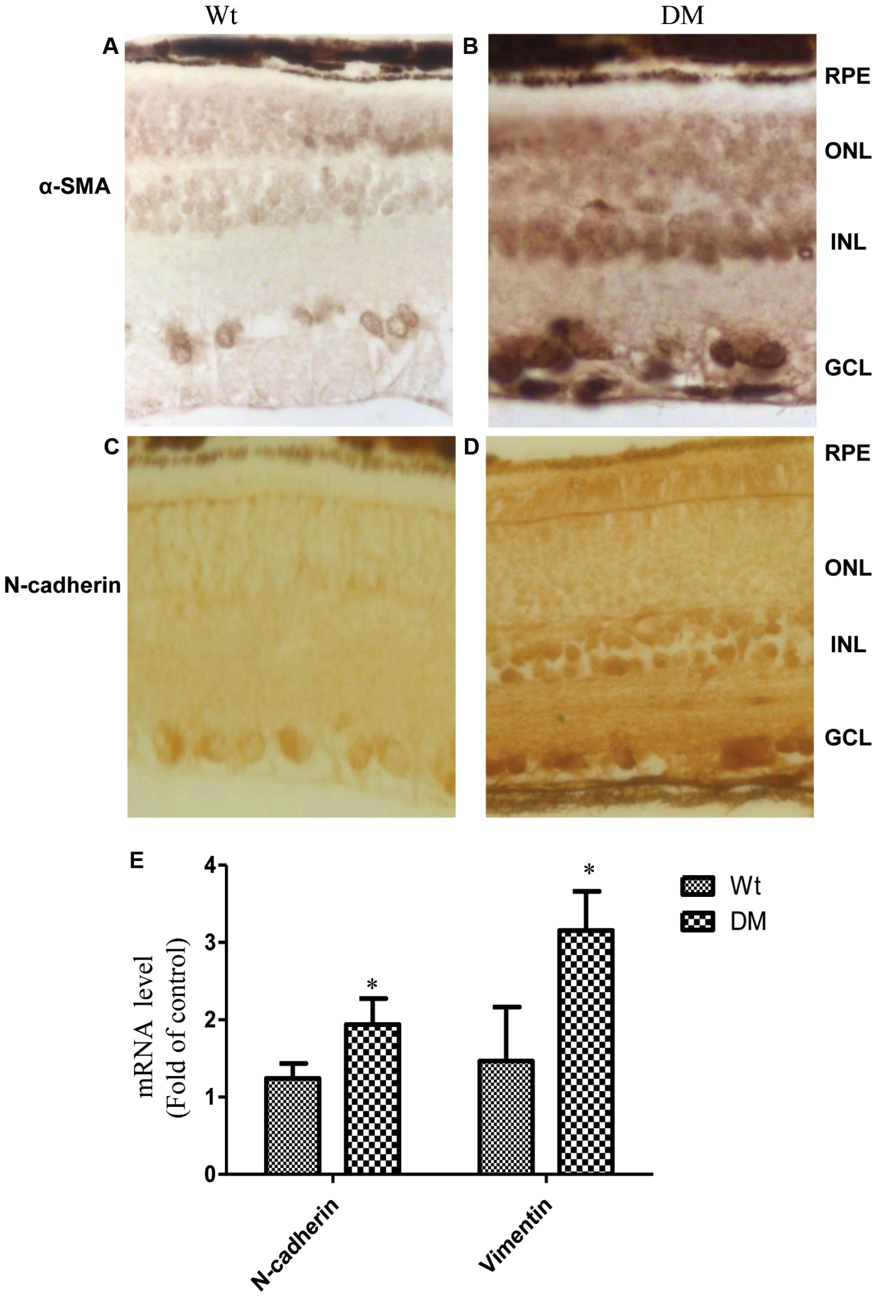

Supplementary Figure S1: Expressions of mesenchymal markers in the retinas of STZ-diabetic rats. (**A–D**) Retinal sections from wild type rats and STZ-diabetic rats were immunostained with the antibodies for α-SMA and N-cadherin. The signal was developed with the diaminobenzidine method (brown color). Representative retinal images showed more intensive signals of α-SMA and N-cadherin in STZ-diabetic rats compared to that of control rats. (**E**) Real time PCR analysis of mRNA levels of N-cadherin and Vimentin in the retinas of Wt and diabetic rats. Values are mean ± s.d., $n = 6$; $*p < 0.05$.
